# Supplementary material for: Hemozoin-catalyzed precipitation polymerization as an assay for malaria diagnosis
Source: Nat Commun. 2019 Mar 25;10:1369. doi: 10.1038/s41467-019-09122-z (PMC6433922; doi:10.1038/s41467-019-09122-z)
Supplement: Supplementary file 1 — Supplementary Information [file 41467_2019_9122_MOESM1_ESM.pdf]

Hemozoin-catalyzed precipitation polymerization as an assay for malaria diagnosis

Omar Rifaie-Graham, Jonas Pollard, Samuel Raccio et al.

Supplementary Information

Supplementary Methods

Materials

Synthetic hemozoin (sHz) was purchased from InvivoGen (San Diego, CA) and was used as received. *N*-isopropyl acrylamide (NIPAAm) was purchased from Sigma Aldrich and was recrystallized from hexane. 2,2′-Azobis(2-methylpropionitrile) (AIBN) was purchased from Sigma Aldrich and was recrystallized from methanol. Deuterated solvents (D_2O, $CDCl_3$) were purchased from Cambridge Isotope Laboratories, Inc. AlbumaxII and RPMI 1664 for *Plasmodium falciparum* culture was obtained from Gibco Life Technologies. Human erythrocytes (AB+) were purchased from Blutspende Zürich. Full blood heparin samples were taken from volunteers at SwissTPH, Basel, and at University of Fribourg. Phosphate buffered saline (PBS) was prepared according to the manual for PBS-tablets from Sigma Aldrich. All other chemicals were purchased from Sigma Aldrich and were used as received. Ultrapure water was obtained from Purelab Flex II (Veolia water system) at 18.2 mΩ using an LC208 purification pack.

Synthesis of PNIPAAm by free radical polymerization

PNIPAAm was synthesized in the absence of solubilized heme to obtain a free-of-heme polymer for UV-vis spectroscopy measurements. 500 mg (4.41 mmol) NIPAAm and 11 mg (67 µmol) AIBN were dissolved in 2 mL of 1,4-dioxane in a 10 mL round bottom flask. The solution was sealed with a septum and bubbled with argon for a period of 1 hour. The reaction was initiated by placing the solution in an oil bath at 90 °C. The reaction was carried out for 6 hours and was quenched by exposing the solution to open air. The resulting polymer was purified by precipitation in hexane. The resulting suspension was centrifuged at 5000 x g for 3 minutes (Thermo Scientific, Heraeus Megafuge 16R, TX-400 x 400 mL Swinging Bucket Rotor). The resulting supernatant was discarded and the pellet was dissolved again. The purification process was repeated 3 times. The polymer was dried overnight *in vacuo* at 40°C.

P. falciparum culture and synchronization

P. falciparum 3D7 parasites were cultured according to standard procedures.[1] Parasites were synchronized during ring stage by incubation in 5% sorbitol at 37°C for 5 minutes.[2]

Preparation of hemozoin from late-stage P. falciparum culture

Natural hemozoin (nHz) from *P. falciparum* 3D7 was obtained from a 30 mL late stage culture with 8 – 15% parasitemia. Hemozoin-containing parasites were enriched using a MACS CS column (Miltenyi Biotec, Bergisch-Gladbach, Germany) in an externally applied magnetic field (vario MACS magnet). The column retained nHz-containing cells because nHz is paramagnetic.[3] After extensive washing (3 culture volumes) with PBS, the column was removed from the magnetic field and eluted with 50 mL PBS. Cells were collected by centrifugation (800 x g, 5 min) and the erythrocytes were selectively lysed by addition of saponin (5 ml of a 0.1 % saponin solution in PBS) for 10 minutes on ice. The suspension was centrifuged. Pellets, which contain intact parasites and cell debris, were resuspended in ultrapure water and frozen at -20°C to hypotonically lyse the parasites. After thawing, the cell lysates were washed 3 times with ultrapure water. The remaining black hemozoin crystals were then dispersed in 1 mL of ultrapure water.

Solubilization of natural and synthetic hemozoin

The dispersion of nHz was centrifuged at 12100 × g for 5 minutes. The supernatant was discarded to obtain a pellet which was dried overnight under vacuum at 40 °C. 3.19 mg of hemozoin were then solubilized in 1 mL of 0.4 M NaOH during 2 h, generating a green solution of solubilized natural hemozoin. The concentration of heme in these nHz stock solutions was quantified by Inductively Coupled Plasma Optical Emission Spectroscopy (ICP-OES).

2.03 mg of sHz were weighted into an Eppendorf tube on a Mettler Toledo XSE balance. 1 mL of 0.4 M NaOH was added. sHz dissolved immediately to yield a green solution. The final concentration of solubilized hemozoin in this stock solution was measured by ICP-OES.

The main difference between nHz and sHz is that the natural hemozoin crystals contain unsaturated lipids, such as linoleic and arachidonic acid and their partly oxidized stages, which are involved in the process of biocrystallisation.[4,5] This difference in composition translates into different solubilities. While it took two hours of incubation in 0.4 M NaOH to completely dissolve nHz, sHz dissolved immediately in this base (See Supplementary Fig. 11 for UV-vis spectra and ref. 6 for more information).

Biocatalytic precipitation polymerization assay for the quantification of hemozoin

Unless otherwise stated in a typical experiment 1.429 g (12.63 mmol) of NIPAAm were weighted together with 250 mg (1.26 mmol) of (+)-sodium L-ascorbate (NaAsc) into a round-bottom flask. Then, 10 mL of sodium phosphate buffer solution (pH 6.5, 0.12 M) were added to obtain a stock solution which was employed for the polymerization reactions. 0.700 mL of this stock solution were pipetted into disposable semi-micro poly(methyl methacrylate) cuvettes (pathlength: 1 cm). Various volumes of hemozoin solutions in 0.4 M NaOH were added and adjusted to a total volume of 50 µL of 0.4 M NaOH. The volume of buffer was adjusted to achieve a total volume of 950 µl in each cuvette. The solutions were then sealed against ambient oxygen by adding 500 µl of mineral oil. The cuvettes were incubated for three minutes at 37 °C. Finally, the polymerization was initiated by the addition of 50 µL of a 375 mM 2-hydroxyethyl 2-bromoisobutyrate (HEBIB) solution in DMF. The final concentration of reagents in the assay was: 18.7 mM HEBIB, 767 mM of NIPAAm and 76.7 mM NaAsc. The cuvette was placed in a thermostated cuvette holder (37 °C) of a UV-vis spectrometer and extinction was recorded at 600 nm or at 380 nm every 30 s with an integration time of 1 s for up to 4 h. The rates of turbidity formation were calculated using a script on Wolfram Mathematica. This script selected two points of the extinction versus time curve (one at 30 % and one at 40 % of the maximum extinction). A line was fitted to these two points to give the effective slope $\Delta E/\Delta t$. The assays were carried out at least in quintuplet. Mean values and standard deviations are reported. These values were then plotted against the concentration of catalyst to obtain a dose-response curve for the polymerization-based hemozoin quantification assay.

Following the work of Hubaux and Vos,[7] using linear regression, two kinds of lower limits may be connected to the notion of confidence limits: the decision limit, and the detection limit. The decision limit represents the lowest signal that can be distinguished from the blank reaction. Above this critical value it is possible to confirm the presence of the analyte in an unknown sample. The

detection limit is the concentration above one can accurately quantify the concentration of the analyte in an unknown sample.

According to literature, the level of parasitemia can be calculated from the hemozoin concentration of a sample.[8,9] An equation to determine the level of parasitemia from the result of the precipitation polymerization assay can be derived as follows: The assay measures the concentration of Hz in a sample $c_{assay}(Hz)$, which allows to calculate the concentration of Hz in blood $c_{blood}(Hz)$:

$$c_{\text{blood}}(Hz) = c_{\text{assay}}(Hz) \cdot \frac{V_{\text{assay}}}{V_{\text{blood}}}\ [ng\ mL^{-1}] \quad (1)$$

With

V_{blood}= volume of blood taken from a patient [µL]

V_{assay}= total volume of assay [µL]

The parasitemia *p* is defined as

$$p = \frac{c(iRBC)}{c_{\text{total}}(RBC)} \cdot 100\ [\%] \quad (2)$$

With

c(iRBC)= concentration of infected red blood cells in blood [mL^{-1}]

$c_{\text{total}}(RBC)$= total concentration of red blood cells in blood [mL^{-1}]

According to Newman et al.,[8] the concentration of hemozoin depends on the concentration of infected red blood cells as follows:

$$c_{\text{blood}}(Hz) = c(iRBC) \cdot m_{\text{max}}(Hz) \cdot a\ [pg\ mL^{-1}] \quad (3)$$

With

$m_{\text{max}}(Hz)$ = mass of hemozoin per infected RBC if all Hb would be converted to Hz [pg]

a= conversion of Hb to Hz [%]

This leads to

$$c(iRBC) = \frac{c_{\text{blood}}(Hz)}{m_{\text{max}}(Hz) \cdot a} \quad (4)$$

$$p = \frac{c_{\text{blood}}(Hz)}{c_{\text{total}}(RBC) \cdot m_{\text{max}}(Hz) \cdot a} \cdot 100\ [\%] \quad (5)$$

$$p = c_{\text{assay}}(Hz) \cdot \frac{V_{\text{assay}}}{V_{\text{blood}}} \cdot \frac{1}{c_{\text{total}}(RBC) \cdot m_{\text{max}}(Hz) \cdot a} \cdot 100\ [\%] \quad (6)$$

The number of red blood cells in one µL of blood is $c_{total}(RBC) = 5 \cdot 10^6\ \mu L^{-1}$.[8]

Newman et al. assumed a mass formation of hemozoin of 0.6 pg per iRBC if 50 % of the haemoglobin was consumed.[8] This gives a value of $m_{\text{max}}(Hz)$= 1.2 pg per iRBC if all hemoglobin would be converted to Hz.

According to Egan et al.[9] *a*≈ 50 % for *P. falciparum* trophozoites. To show that the assay can detect hemozoin in the trophozoite stage, we chose *a*= 0.50.

With these assumptions and V_{blood}= 1000 µL and V_{assay}= 1000 µL, the parasitemia can be calculated as

$$p = c_{\mathrm{assay}}(Hz) \cdot 1.28205 \cdot 10^{-6}\ \frac{\mu L}{pg}\ [\%] \qquad (7)$$

Limit of detection of the precipitation polymerization assay: The limit of detection of the assay is $c_{\mathrm{LoD}}(Hz) = 0.846$ ng mL^{-1}. According to equation (7), this corresponds to $p_{\mathrm{LoD}} = 2.82 \cdot 10^{-5}\%$.

$$c_{LoD}(iRBC) = \frac{p_{\mathrm{LoD}} \cdot c_{\mathrm{total}}(RBC)}{100} \qquad (8)$$

With $c_{\mathrm{total}}(RBC) = 5 \cdot 10^{6}\ \mu L^{-1}$

$$c_{\mathrm{LoD}}(iRBC) = 1.41\ \mu L^{-1} \qquad (9)$$

To demonstrate that the test can be miniaturized, polymerization mixtures were prepared in cuvettes according to the method reported above. After introduction of the initiator, the liquid was sucked into glass capillaries (BLAUBRAND micropipettes, intraMark, 100 µL; BRAND). The capillaries were sealed with bee wax and incubated at 37°C in an incubator (INCUCELL, MMM Medcenter Einrichtungen) for one hour. Then, the capillaries were imaged with a HTC ONE M8s digital camera (HTC UltraPixel™ camera, BSI sensor, pixel size 2.0 um, sensor size 1/3", f/2.0, 28mm lens) mounted to a Stemi DV4 Stereomicroscope (Carl Zeiss, Jena).

Determination of hemozoin concentration by ICP-OES

Inductively Coupled Plasma Optical Emission Spectroscopy (ICP-OES, PerkinElmer Optima 7000 DV) was used to determine the concentration of solubilized nHz and sHz in their stock solutions. To this end, 100 µL of a stock solution in 0.4 M NaOH were transferred into a 15 mL falcon tube and diluted with ultrapure water to a total volume of 6 mL. The sample was then measured by ICP-OES (wavelength used: 238.2 nm for Fe) and its iron content determined with the help of a calibration curve, obtained by measuring aqueous solutions (ultrapure water) with known concentrations of iron standard for ICP (TraceCERT, 1000 mg L^{-1} Fe in nitric acid; Sigma-Aldrich). The stock solution of nHz was determined to have a concentration of 177 mg L^{-1} which corresponds to 0.29 mM with respect to heme. The stock solution of sHz was determined to have a concentration of 1991 mg L^{-1} which corresponds to 3.2 mM with respect to heme.

Isolation of hemozoin from parasite-spiked blood samples

<u>Full isolation of the biocrystal</u>: 10 mL of freshly taken human blood was collected in heparin-coated tubes (Sarstedt Monovette Li-Heparin LH/9 mL). 1 mL of the blood was transferred into 15 mL Falcon tubes (polypropylene) and spiked with known quantities of a *P. falciparum* non-synchronized culture (concentration of infected red blood cells determined by Giemsa staining). The RBC membranes were selectively lysed with 14 mL of a 0.3 % (w/v) saponin solution in PBS. The lysate was vortexed and centrifuged at 5000 x g for 10 min. The supernatant was removed and the pellet was washed twice with PBS (15 mL). Then, the parasite membranes were lysed with 15 mL of a 5 % (w/v) SDS in ultrapure water, followed by a centrifugation at 5000 x g for 120 min. In a following step, the pellets were washed twice with 15 mL of ultrapure water. The pellets were then dried overnight in vacuum oven at 70 °C. To dissolve the pellet, 55 µL of a 0.1 % (w/v) SDS in 0.4 M NaOH aqueous solution were

then added and sonicated for 20 min. After a centrifugation (5000 x g, 10 min), 50 µL of this solution were introduced into the polymerization assay.

<u>*Parasite isolation by size exclusion chromatography*</u>: 10 mL of human blood were collected in heparin-coated tubes (Sarstedt Monovette Li-Heparin LH/9 mL). 50 µL of this sample were transferred into a 1.5 mL Eppendorf tube (polypropylene) and spiked with known quantities of a non-synchronized *P. falciparum* culture (concentration of parasites determined by Giemsa staining). The RBC membranes were selectively lysed with 250 µL of a 0.3 % (w/v) solution of saponin in PBS. The lysate was submitted to a size exclusion column consisting of Sepharose 4B CL packed in a Biotage SNAP Ultra 10 g column as the stationary phase with PBS as the mobile phase. Prior to the chromatography, the columns were equilibrated by substituting the 20:80 ethanol/water solution in which the stationary phase is delivered with 50 mL of ultrapure water and then 50 mL of PBS buffer. Then, the lysate was loaded to the column and eluted by passing 6 mL PBS through the column by means of a hand-driven syringe., The first 5 mL of PBS were collected and contained the parasites. A clear band for the presence of hemoglobin was observed and remained on the column, which was afterwards discarded. The isolate was centrifuged in a 15 mL Falcon tube at 5000 x g for 10 min forming a pellet. The supernatant was discarded and the pellet was dried overnight in a vacuum oven at 60 °C. The parasites were further lyzed and their contents solubilized in 55 µL of a 0.4 M NaOH aqueous solution and sonicated for 30 min (sonicator bath Sonoswiss SW3). The sample was then centrifuged for 10 min at 5000 x g to spin down the sample and 50 µL were introduced into the polymerization assay.

Preparation of purified PNIPAAm samples for GPC, UV-Vis and ^{1}H-NMR

Polymerization reactions were carried with 60 µg mL^{-1} of sHz. After a reaction time of 1 hour, the polymerization reactions were placed in the freezer and frozen at -20 °C. The oil layer was then removed by means of a pipette. The solution was transferred from the cuvette to an Eppendorf tube. The tube was heated to 60°C and centrifuged for 3 minutes. The supernatant was removed and the polymer was dissolved in 1 mL of THF, followed by a precipitation in 40 mL of hexane. The polymer was separated by centrifugation. The precipitation of the polymer in hexane was repeated three more times. Finally, the polymer was dried overnight in a vacuum oven at 40°C. 1 mg of the polymer was redissolved in 1 mL DMF and analyzed by GPC. 10 mg were redissolved in 0.8 mL of D_2O and analyzed by ^{1}H-NMR.

Determination of conversion by ^{1}H-NMR

Polymerization reactions were carried out with 1 µg mL^{-1} sHz and with 60 µg mL^{-1} sHz. After a reaction time of 2 000 seconds the samples were allowed to cool to room temperature so that the PNIPAAm would dissolve. Then, 100 µL of each reaction were taken and diluted in 700 µL of D_2O. The solution was mixed and transferred into an NMR tube for analysis.

The conversion was calculated by the following equation (10):

$$Conversion = \frac{b' + c'}{a + b + b' + c'} \qquad (10)$$

With: a + b, integral of the peak corresponding to the two hydrogen atoms of the vinyl group of the monomer (6.12 – 6.33 ppm); b' + c', with the integral of the peaks corresponding to two hydrogen atoms per repeat unit in the polymer backbone (1.38 – 1.90 ppm).

The proton assignation and integrals are shown in the supplementary Fig. 5.

Instrumentation

UV-Vis measurements were carried out on an Analytik Jena Specord 50 Plus spectrophotometer with a 6-cell changer thermostated at 37 °C with a Julabo heating circulator TD-6. ^{1}H-NMR measurements were recorded with a Bruker Avance III 300 MHz NMR spectrometer. ^{1}H-NMR spectra were referenced internally with residual solvent peaks. GPC traces were recorded on an Agilent Viscotec mixed system with a 1100 series pump and autosampler connected to a TDA 305 (refractive Index, variable wavelength detector), viscometer and 90 ° light scatterer), equipped with a series of gram columns (pre-column (5 cm), 30 Å (30 cm) and two 1000 Å (30 cm), all 10 µm particles and 0.8 cm in diameter, PSS). The detector and columns were run at 60 °C with DMF (20 mM LiBr) as eluent at 1 mL min^{-1}. 100 µL samples were injected. The system was calibrated with narrowly distributed poly(methyl methacrylate) standards from Sigma-Aldrich. Light scattering data were collected at constant temperature (37 °C) using a commercial goniometer instrument (3D LS Spectrometer, LS Instruments AG, Switzerland). The primary beam was formed by a linearly polarized and collimated laser beam (Cobolt 05-01 diode pumped solid state laser, λ = 660 nm, P max. = 500 mW), and the scattered light was collected by two single-mode optical fibers equipped with integrated collimation optics. The collected light was coupled into two high-sensitivity APD detectors via laser-line filters (Perkin Elmer, Single Photon Counting Module), and their outputs were fed into a two-channel multiple-tau correlator. To suppress the influence of multiple scattering events on the auto-correlation spectra, these two channels were cross-correlated. The field auto-correlation functions were obtained via the Siegert relation: $g_1(t) = \sqrt{g_2(t) - 1}$ where $g_2(t)$ is the intensity auto-correlation function constructed from the temporal fluctuations of the depolarized component of the scattered intensity. Regarding fitting, it is worthwhile to point out that $g_1(t)$ is always expected to be a monotonic decreasing function of time, since $\partial_t\, g_1(t) < 0$. Furthermore, $g_1(t)$ is also expected to be a convex function, since $\partial_t^2\, g_1(t) > 0$. A sequence of correlation data that did not satisfy these general properties were regarded as being artefacts and were discarded without further analysis. Correlation functions were recorded during 30-second long consecutive intervals. Each correlation function was either analyzed or discarded by following the criteria described above. From the analysis, the average intensity-weighted hydrodynamic radius was obtained via the Stokes-Einstein relation. For better clarity, consecutive results were averaged and the uncertainty was expressed by the standard deviation. Typically, we averaged 10 consecutive results corresponding to an interval of 300s, that is, from e.g. 100 correlation function analyzed, we obtained in the end 10 nearly equally spaced (equidistant in time) data pairs described by the average and standard deviation.

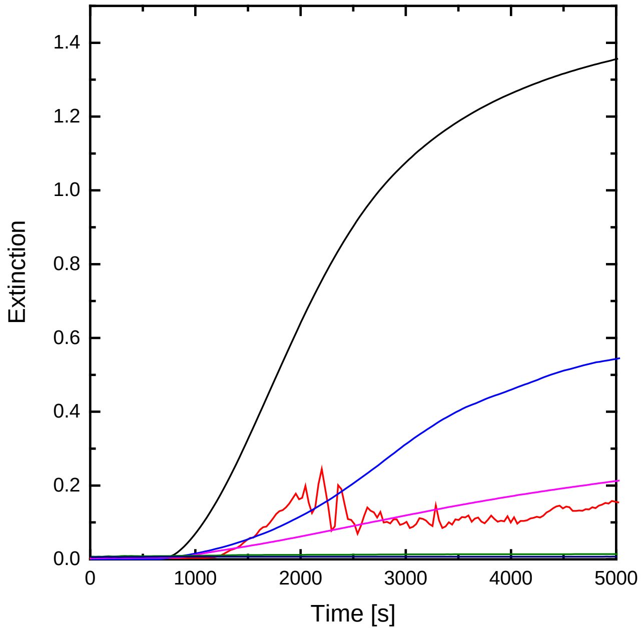

Supplementary Figure 1 | Control reactions with 100 ng mL^{-1} sHz show all ingredients of the assay are necessary to achieve a good read-out. Standard assay (black); assay without oil layer (red); assay without initiator (blue); assay without catalyst (pink); assay without reducing agent (green) and assay without monomer (purple).

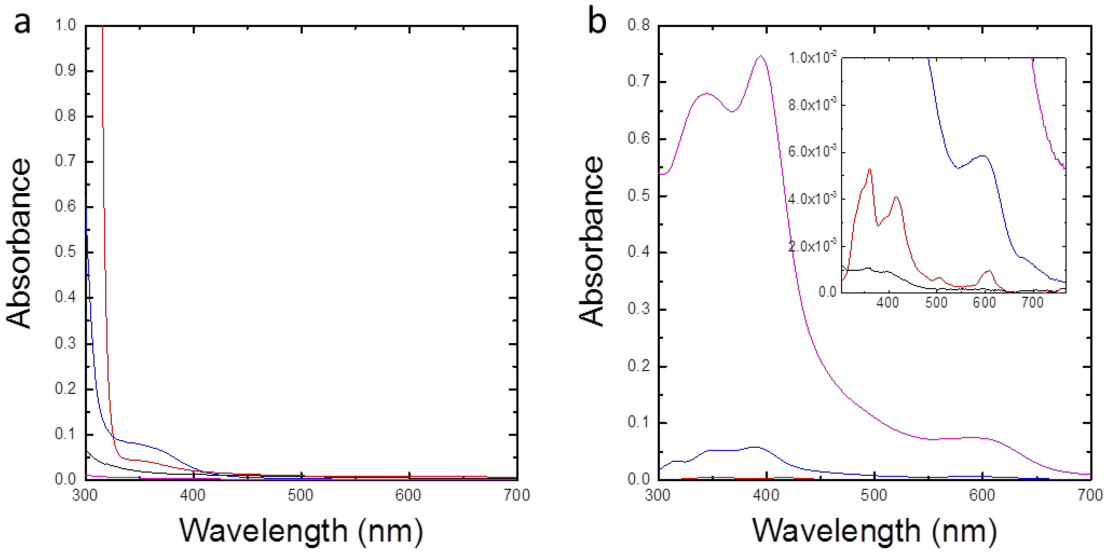

Supplementary Figure 2 | UV-vis spectra of the reagents of the assay. a, UV-vis spectra of NIPAAm (blue), sodium ascorbate (red), HEBIB (black) and PNIPAAm (pink) at the concentrations of the assay in 0.9 mL of 0.1 M phosphate buffer pH 6, 50 µL of 0.4 M NaOH and 50 µL of DMF. **b,** UV-vis spectra of sHz dissolved in 0.4 M NaOH. c (sHz)= 10 ng mL^{-1} (black), 100 ng mL^{-1} (red), 1000 ng mL^{-1} (blue) and 10000 ng mL^{-1} (pink). The inset shows an expansion of the y-axis.

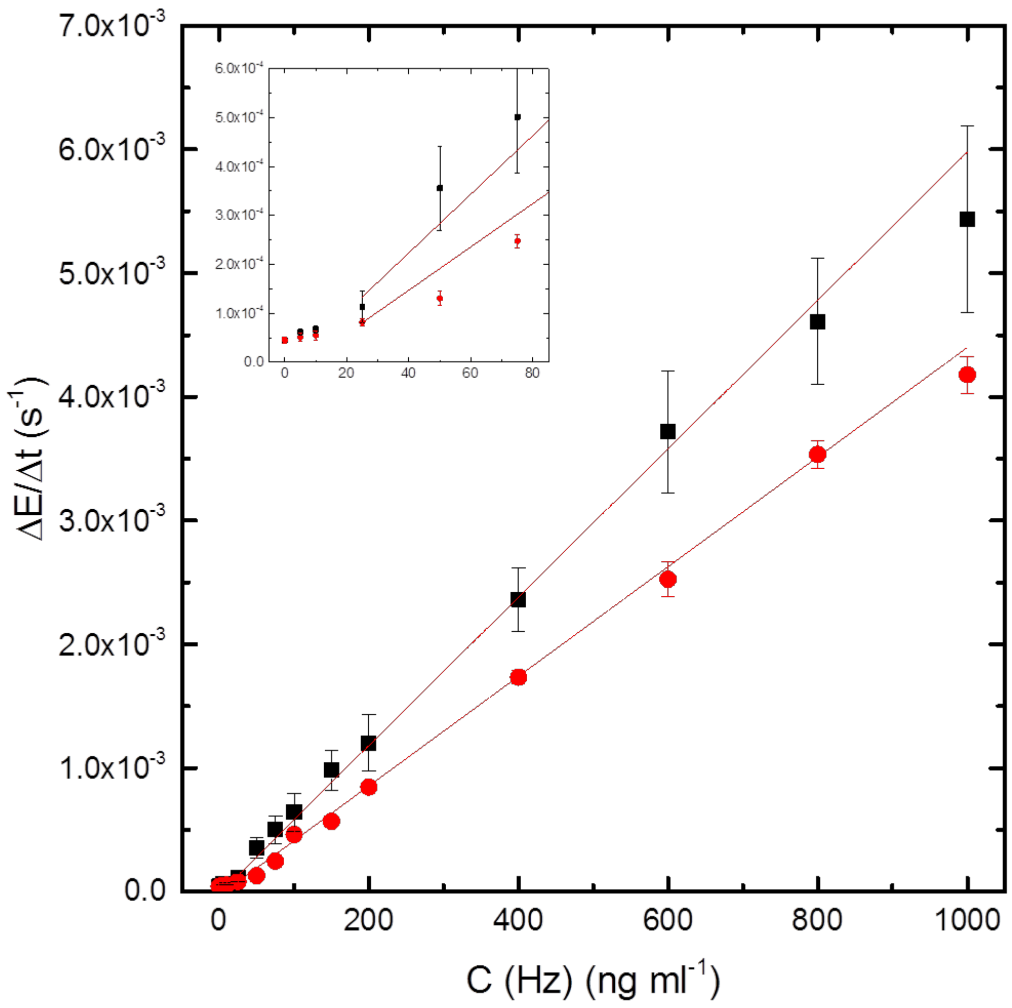

Supplementary Figure 3 | Dose-response curves of sHz (black) and nHz (red) at 600 nm. The data of nHz was repeated from Fig. 2c of the main manuscript to allow a direct comparison of the data sets. (n = 5, average and SD are reported)

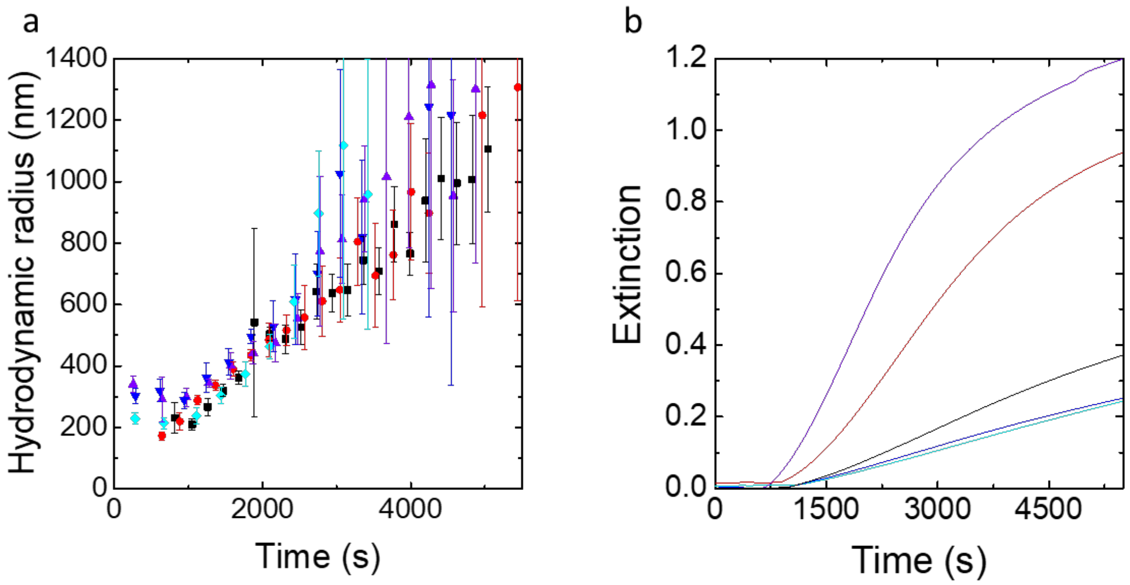

Supplementary Figure 4 | Comparison of dynamic light scattering and extinction measurements during Hz-catalyzed precipitation polymerizations of NIPAAm. a, Dynamic light scattering analysis of particle size for polymerizations catalyzed by 5 ng mL^{-1} (light blue), 10 ng mL^{-1} (dark blue), 25 ng mL^{-1} sHz (black; same data set as reported in Figure 3a), 50 ng mL^{-1} (red) and 75 ng mL^{-1} (purple) sHz (mean values determined as described in the experimental section, SD). **b**, Time-dependent extinction measurements at 600 nm for polymerizations catalyzed by 5 ng mL^{-1} (light blue), 10 ng mL^{-1} (dark blue), 25 ng mL^{-1} (black), 50 ng mL^{-1} (red) and 75 ng mL^{-1} (purple) sHz.

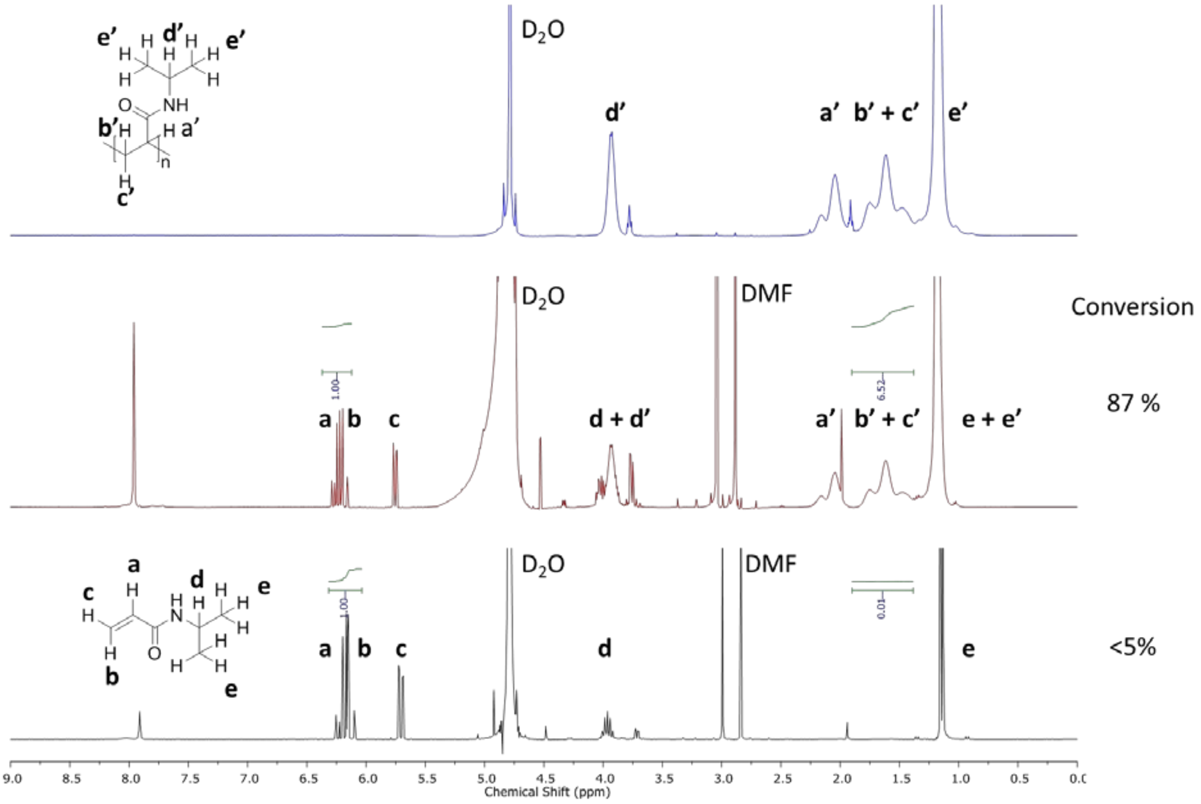

Supplementary Figure 5 | ^{1}H NMR spectra for the determination of monomer to polymer conversion. ^{1}H NMR spectra of the reaction mixture after 2000 seconds. At this stage, the polymerization stopped and encountered the plateau phase of turbidity. The concentration of solubilized sHz was of 1 μg mL^{-1} (black) and 60 μg mL^{-1} (red). As a comparison, a ^{1}H NMR spectrum of the purified polymer (blue) obtained with a catalyst concentration of 60 μg mL^{-1} is shown. At a sHz

concentration of 1 µg mL^{-1}, the conversion is too low to be determined by the NMR. Hence we assume that the conversion is lower than 5% as this corresponds to the detection limit of NMR.

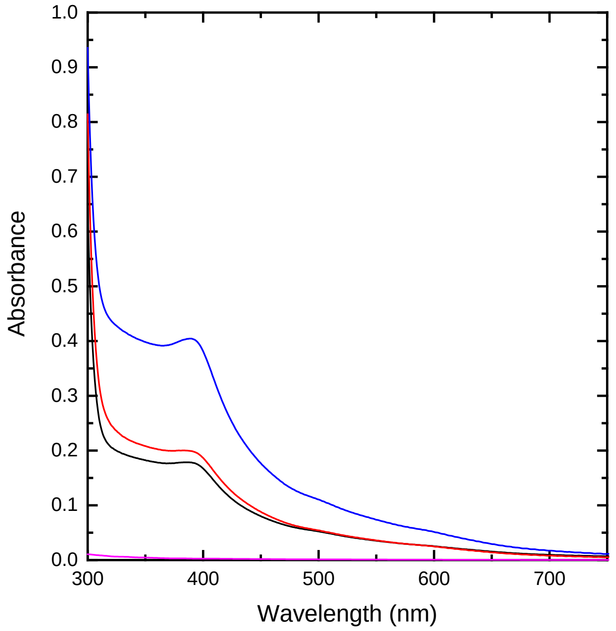

Supplementary Figure 6 | UV-vis spectra of purified PNIPAAm polymers. Polymers were produced in assays with 10 µg mL^{-1} (black), 15 µg mL^{-1} (red) and 20 µg mL^{-1} (blue) sHz. The polymers were purified after a reaction time of 1 h as described in the experimental section. The purified polymers have absorption bands at 387 nm, which indicate the presence of heme. PNIPAAm was also synthesized by free radical polymerization in the absence of heme as a means of comparison (pink).

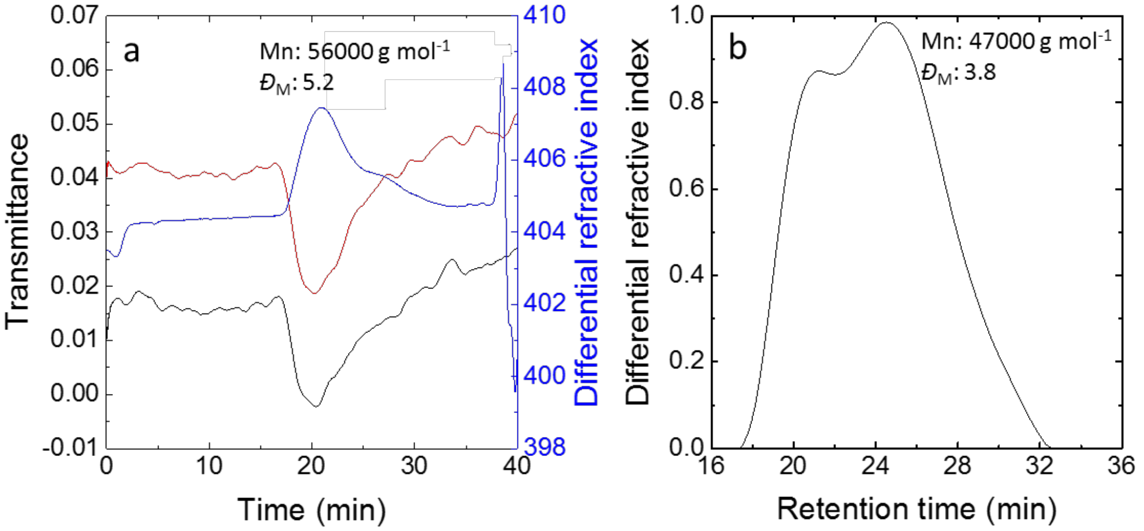

Supplementary Figure 7 | Gel permeation chromatograms of purified PNIPAAm obtained by Hz-catalyzed precipitation polymerization. a, GPC trace of a polymer that was synthesized with 60 µg mL^{-1} of sHz and purified as described in the experimental section after a reaction time of 1h. UV absorbance at 387 nm (red) and 421 nm (black), as well as RI trace (blue) of the chromatogram are shown. **b**, GPC trace of a polymer synthesized with 1 µg mL^{-1} sHz and purified as described in the experimental section after a reaction time of 1h. The RI trace is shown.

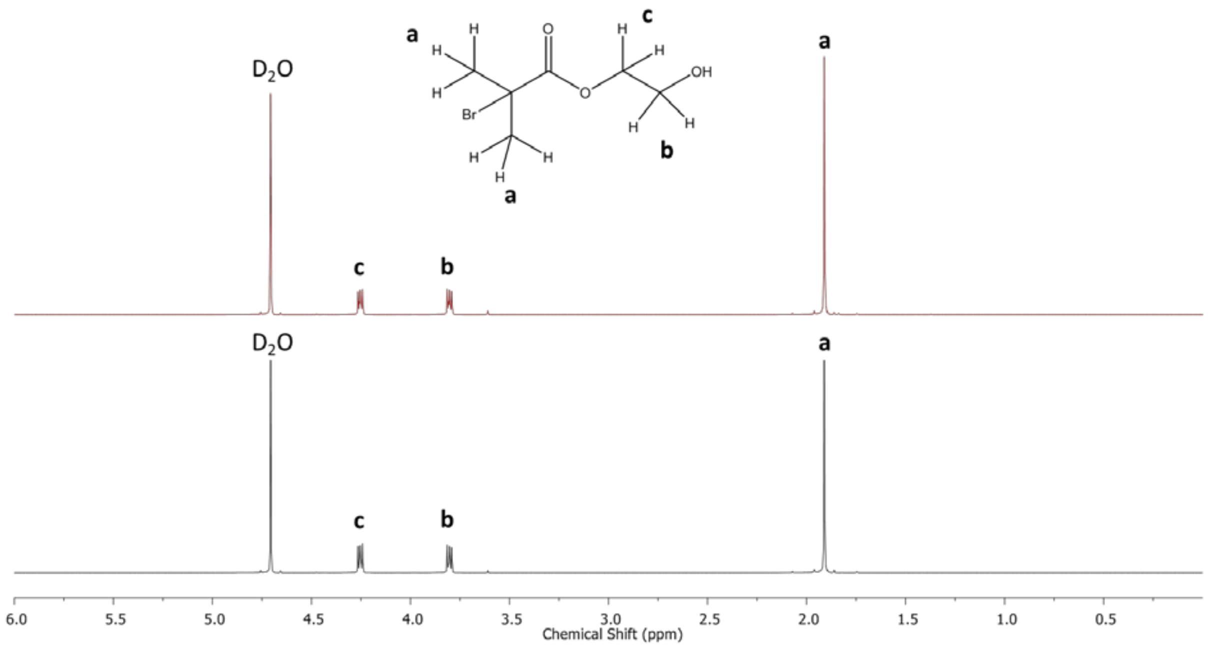

Supplementary Figure 8│ ^{1}H NMR spectra of HEBIB before (black) and after storage for 2 months at 50°C (red).

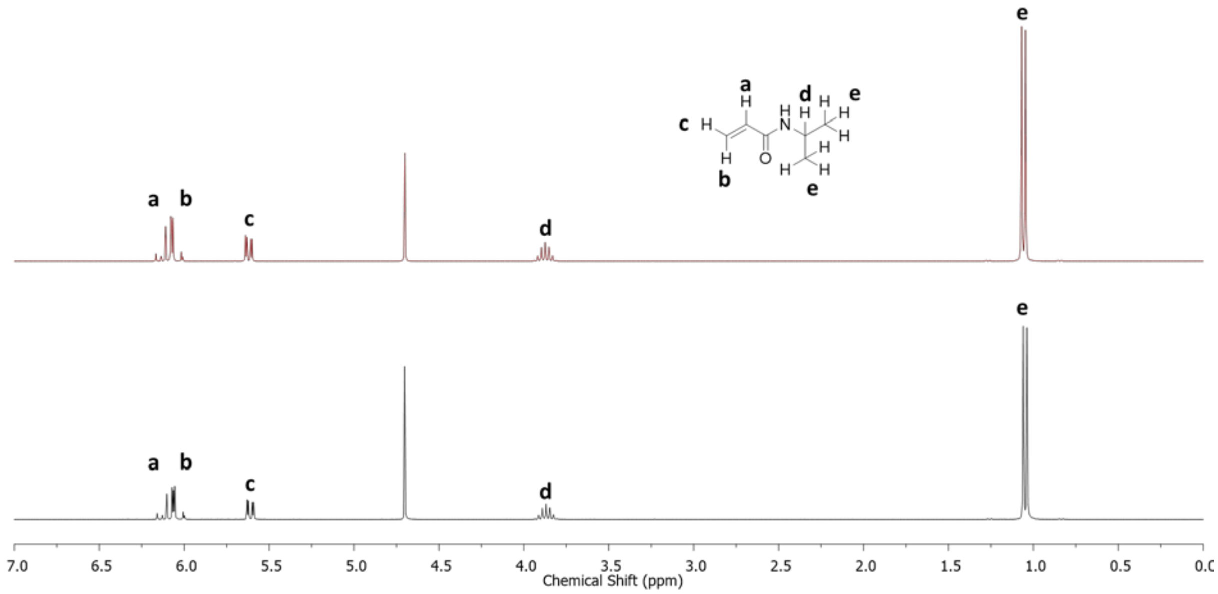

Supplementary Figure 9 │ ^{1}H NMR spectra of NIPAAm before (black) and after storage for 2 months at 50°C (red).

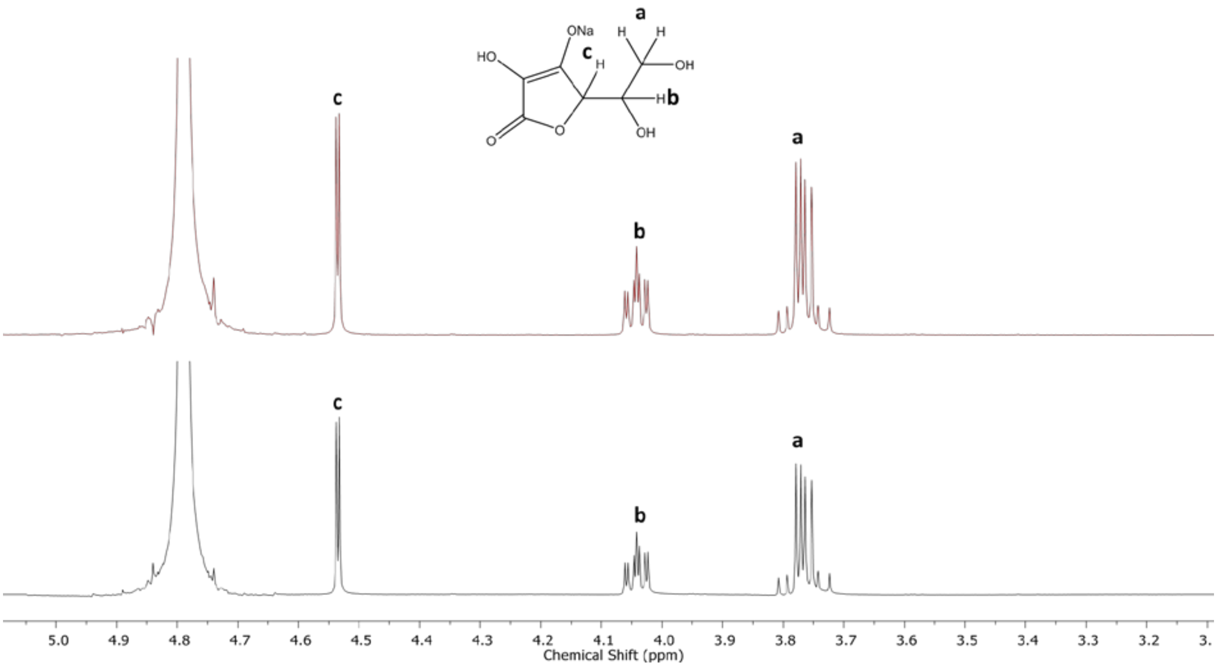

Supplementary Figure 10 | ^{1}H NMR spectra of NaAsc before (black) and after 2 months of storage at 50°C (red).

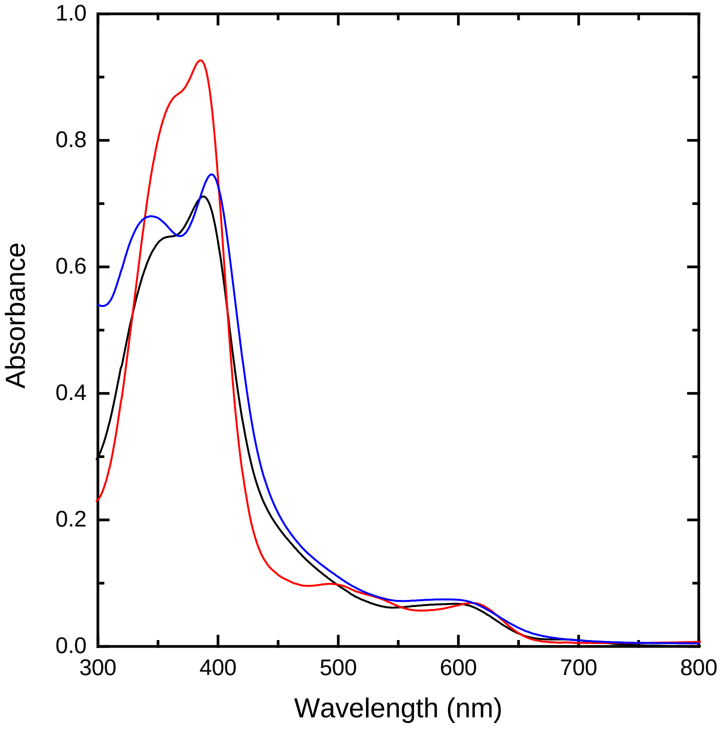

Supplementary Figure 11 | UV-vis spectra of 1.62 µM natural hemozoin (blue), 1.62 µM synthetic hemozoin (black) and 1.53 µM hemin (red) in 0.4 M NaOH.

Supplementary References

1 Moll, K., Ljungström, I., Perlmann, H., Scherf, A. & Wahlgren, M. (MR4/ATCC, Manassas, VA, USA, 2008).

2 Lambros, C. & Vanderberg, J. P. Synchronization of Plasmodium falciparum Erythrocytic Stages in Culture. *J. Parasitol.* **65**, 418-420 (1979).

3 Newman, D. M., Matelon, R. J., Wears, M. L. & Savage, L. B. The in vivo diagnosis of malaria: feasibility study into a magneto-optic fingertip probe. *IEEE J. Sel. Top. Quantum Electron.* **16**, 573-580 (2010).

4 Schwarzer, E., Kühn, H., Valente, E. & Arese, P. Malaria-parasitized erythrocytes and hemozoin nonenzymatically generate large amounts of hydroxy fatty acids that inhibit monocyte functions. *Blood* **101**, 722-728 (2003).

5 Coronado, L. M., Nadovich, C. T. & Spadafora, C. Malarial hemozoin: From target to tool. *Biochim. Biophys. Acta, Gen. Subj.* **1840**, 2032-2041 (2014).

6 Asher, C., de Villiers, K. A. & Egan, T. J. Speciation of ferriprotoporphyrin IX in aqueous and mixed aqueous solution is controlled by solvent identity, pH, and salt concentration. *Inorg. Chem.* **48**, 7994-8003 (2009).

7 Hubaux, A. & Vos, G. Decision and detection limits for calibration curves. *Anal. Chem.* **42**, 849-855 (1970).

8 Newman, D. M. *et al.* A Magneto-Optic Route toward the In Vivo Diagnosis of Malaria: Preliminary Results and Preclinical Trial Data. *Biophys. J.* **95**, 994-1000 (2008).

9 Egan, T. J. *et al.* Fate of haem iron in the malaria parasite Plasmodium falciparum. *Biochem. J* **365**, 343-347 (2002).
