# Supplementary material for: Hemozoin-catalyzed precipitation polymerization as an assay for malaria diagnosis
Source: Nat Commun. 2019 Mar 25;10:1369. doi: 10.1038/s41467-019-09122-z (PMC6433922; doi:10.1038/s41467-019-09122-z)
Supplement: Supplementary file 3 — Description of Additional Supplementary Files [file 41467_2019_9122_MOESM3_ESM.pdf]

Description of Additional Supplementary Files

File Name: Supplementary Movie 1
Description: Precipitation polymerizations of N-isopropylacrylamide at 37°C as an assay to detect and quantify the malaria biomarker hemozoin. "A" was a control experiment that did not contain hemozoin. The concentration of synthetic hemozoin (sHz) in "B" corresponded to 400 ng mL^{-1}, "C" contained 800 ng mL^{-1} and "D" 1200 ng mL^{-1} sHz. Upon addition of HEBIB in DMF, no reaction was observed in the control cuvette. The formation of turbidity was faster with higher concentrations of sHz which acted as catalyst. The back of the cuvette was colored in black to achieve a better contrast between transparent solution and turbid suspension.
